# Efficacy of Dulaglutide in a Patient With Type 2 Diabetes, High Cardiovascular Risk, and HIV: A Case Report

**DOI:** 10.3389/fendo.2022.847778

**Published:** 2022-02-28

**Authors:** Angela Dardano, Michele Aragona, Giuseppe Daniele, Roberto Miccoli, Stefano Del Prato

**Affiliations:** ^1^Department of Clinical & Experimental Medicine, University of Pisa, Pisa, Italy; ^2^Section of Metabolic Diseases & Diabetes, Azienda Ospedaliero Universitaria Pisana, Pisa, Italy

**Keywords:** case report, GLP-1 receptor agonist, dulaglutide, type 2 diabetes, ASCVD, HIV infection

## Abstract

**Background:**

Type 2 diabetes (T2D) is a common comorbidity in people living with HIV (PLWH). Anti-hyperglycemic treatment in PLWH is still a challenge, and no randomized controlled studies using new glucose-lowering agents are currently available.

**Case Description:**

A 55-year-old-women was admitted to our Diabetes Unit because of hyperosmolar hyperglycemic state (HHS) and sepsis. The medical history included HIV infection and insulin-treated diabetes. On clinical examination, the lady appeared dehydrated with dry buccal mucosa, tachycardia, altered mental status, genital infection, and fever. On admission, plasma glucose was 54.5 mmol/L, HbA1c 155 mmol/mol, osmolarity 389.4 mOsm/kg, bicarbonate 24.6 mmol/L with no detectable serum ketones. The patient was treated with i.v. fluid and insulin, and antibiotic therapy commenced. Upon HHS and sepsis resolution, a basal-bolus insulin therapy was implemented that was followed by significant improvement of daily glucose profiles and progressive reduction of insulin requirement until complete discontinuation. A low dose of metformin plus linagliptin was started. Since a severe atherosclerotic disease was diagnosed, a GLP-1 receptor agonist, dulaglutide, was added to metformin upon linagliptin withdrawal with maintenance of good glycemic control, treatment adherence and amelioration of quality of life and no side effects.

**Conclusion:**

This case suggests that GLP-1 receptor agonist therapy may be effective and safe for treatment of T2D with high cardiovascular risk in PLWH, supporting the need of clinical trials directly assessing the safety and the efficacy of GLP-1 receptor agonist in these individuals.

## Introduction

In the last few years, introduction of antiretroviral therapy (ART) has much improved treatment of people living with HIV (PLWH) (1). Atherosclerotic cardiovascular disease (ASCVD) remains a main cause of morbidity and mortality among PLWH (2). Rates of myocardial infarction, heart failure, stroke, and other cardiovascular diseases (CVD) are greater in PLWH than in uninfected subjects (3). Moreover, PLWH are also more likely to develop type 2 diabetes (T2D) than people without HIV (4, 5) often due to diabetogenic effects of anti-HIV medications (6, 7). Currently, the management of T2D in people with HIV is still a matter of debate (8) and no dedicated trials have addressed the potential of novel anti-hyperglycemic agents.

We herein report the successful use of dulaglutide, a glucagon-like peptide-1 receptor agonist (GLP-1 RA), in a patient with T2D, HIV, and high atherosclerotic risk.

## Case Description

**Figure 1:**
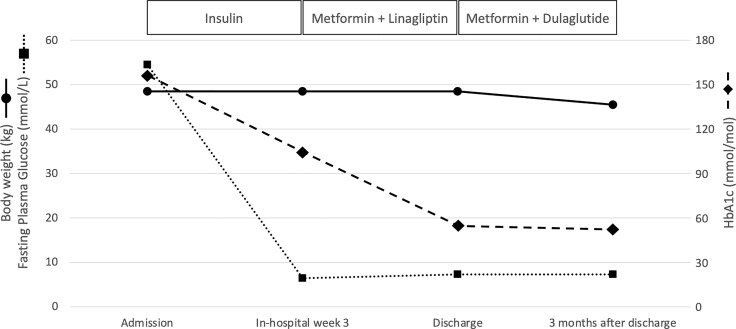
Effects of antihyperglycemic therapy on fasting plasma glucose, HbA1c and body weight.

In April 2021, a 55-year-old woman was admitted to our Diabetes Unit because of hyperosmolar hyperglycemic state (HHS) and sepsis. Prior medical history included HIV infection, insulin-treated diabetes mellitus (both diagnosed at age 42), hypercholesterolemia, non-proliferative diabetic retinopathy, and chronic kidney disease. Home therapy included a three-drug combination ART (dolutegravir 50 mg, abacavir 600 mg, lamivudine 300 mg), arbitrarily stopped due to intolerance, atorvastatin (10 mg) and insulin therapy with poor adherence to treatment due to fear of hypoglycemia. On clinical examination, patient appeared dehydrated with dry buccal mucosa, tachycardia (120 beats per minute), altered mental status, fever (38.3°C), and ulcerated vulvar lichen sclerosus with signs of genital infection and purulent secretions. Her body weight was 48.5 kg (BMI 20.2 kg/m^2^). The Malnutrition Universal Screening Tool (MUST) excluded malnutrition, undernutrition, or weight loss ≥5% in past 3-6 months. No drug addiction was apparent. Systolic blood pressure was 155 mmHg and diastolic blood pressure 90 mmHg. On admission, plasma glucose level was 54.5 mmol/L, HbA1c 155 mmol/mol, osmolarity 389.4 mOsm/kg, bicarbonate 24.6 mmol/L, and there were no detectable serum ketones (**Table 1**). Lipid levels were: total cholesterol 2.09 mmol/L; LDLc 0.57 mmol/L; HDLc 0.65 mmol/L, and triglycerides 1.51 mmol/L. Real time PCR confirmed the HIV-1 genoma with high HIV viral load (68.900 copies/ml). CD4 cell count was 362.6/μL (normal values: 410-1590/μL). Blood cultures were positive for methicillin-susceptible Staphylococcus aureus (MMSA). The patient was treated with i.v. fluid and insulin infusion and started on an empirical antibiotic therapy (piperacillin/tazobactam) that was subsequently switched to target MMSA (daptomycin and oxacillin). A transesophageal echocardiography excluded infective endocarditis. Upon HHS and sepsis resolution, basal-bolus insulin therapy was initiated yielding significant improvement of daily glucose profiles and progressive reduction of insulin requirement. Three weeks after initiation of insulin therapy, total daily insulin requirement was 0.3 unit per kg of body weight (approximately 15 U/day) with fasting capillary glucose levels ranging between 5.55-6.44 mmol/L and postprandial ones between 8.32-9.44 mmol/L with no hypoglycemic events. Pancreatic beta cell reserve was preserved as indicated by a fasting plasma C-peptide level of 0.5761 nmol/L. The search for anti-GAD and anti-IA2 autoantibodies was negative supporting a diagnosis of type 2 diabetes. **Table 1** shows changes in laboratory parameters at hospital admission and 21 days later when insulin therapy was stopped and low dose of metformin (500 mg twice daily) plus linagliptin (5 mg once daily) were started. On a vascular screening, bilateral carotid artery stenosis (50% in the left bulbar internal carotid artery) and bilateral hemodynamically significant renal artery stenosis (> 80% in the para-ostial district of the left renal artery and > 60% on the right) were identified. Magnetic resonance imaging of the brain showed chronic ischemic vasculopathy of the semi-oval centers and radiate crowns. An abdominal ultrasound (US) examination showed liver enlargement with rounded margins and inhomogeneous distribution of steatosis. In view of the patient’s high cardiovascular risk linagliptin was discontinued and GLP-1 receptor agonist therapy in combination with metformin was initiated in agreement with current guidelines (9, 10). At discharge, the complete treatment plan for the patient included dulaglutide (at starting dose of 0.75 mg once weekly), metformin (adjusted to 500 mg once daily to prevent pharmacological interaction with antiretroviral therapy), atorvastatin (10 mg once daily), aspirin (100 mg once daily), bisoprolol (1.25 mg once daily), amlodipine (5 mg once daily), and bictegravir/emtricitabine/tenofovir alafenamide (50 mg/200 mg/25 mg daily), as prescribed by the local infectious disease specialist, given the patient intolerance to previous antiretroviral therapy. Three months after starting dulaglutide, HbA1c was 51 mmol/mol, fasting glucose 7.3 mmol/L and self-monitoring of blood glucose showed a good glycemic control. The patient reported no hypoglycemia nor gastro-intestinal side effects ensuring a high level of adherence to therapy; body weight dropped from 48.5 kg to 45.5 kg with a final BMI of 18.9 Kg/m^2^. At follow-up, an abdominal US no longer detected liver steatosis, although within the limit of inter-operator variability. Urinary albumin-to-creatinine ratio (from 346.7 to 92.1 mg/g) and creatinine value (from 129.9 to 96.4 μmol/L) improved as well and the HIV viral load was markedly reduced (29 copies/ml). **Figure 1** summarizes the effects of antihyperglycemic therapy on fasting plasma glucose, HbA1c and body weight.

**Table 1:** Lab test on admission and 21 day hospitalization.

	On admission	Day 21	Normal values
**Fasting plasma glucose (mmol/L)**	54.5	6.44	3.3-5.5 mmol/L
**HbA1c (mmol/mol)**	155	105	20-30 mmol/mol
**Creatinine (μmol/L)**	129.9	125.6	44-96.8 μmol/L
eGFR* (ml/min/1.73m^2^)	38.8	41.5	> 90 ml/min/1.73m^2^
**Osmolality (mOsm/kg)**	389.4	290.5	280-300 mOsm/kg
**Sodium (mmol/L)**	152	135	135-145 mmol/L
**Bicarbonate (mmol/L)**	24.6	27.2	22-30 mmol/L
**Potassium (mmol/L)**	4.01	3.82	3.4-4-5 mmol/L
**Urea (mmol/L)**	22.6	6.5	3.57-17.85 mmol/L
**Blood urea nitrogen (mmol/L)**	22.85	6.43	< 8.21 mmol/L
**Chloride (mmol/L)**	109	98	98-107 mmol/L
**Calcium (mmol/L)**	2.69	2.31	2.15- 2.55 mmol/L
**C-reactive protein (mg/L)**	312.1	37.1	< 5 mg/L
**Procalcitonin (ng/L)**	5080	80	< 500 ng/L

*CKD-EPI.

## Methods

Plasma glucose was measured by the glucose oxidase reaction (Glucose Oxidase Analyzer). Plasma C-peptide was measured by a radioimmunoassay (Pantec Srl Turin, Italy). Anti-GAD and anti-IA2 autoantibodies were analyzed by a radioimmunoassay using a commercial kit (Medipan, Berlin, Germany). All other parameters were determined according to standard methods. Clinical laboratory data are reported in Standard International units. Reference values for healthy adults in our Laboratory are reported in **Table 1**.

## Discussion

To the best of our knowledge, the literature on the use of the newer classes of anti-hyperglycemic agents (i.e., sodium-glucose cotransporter 2 inhibitors, SGLT2i and GLP-1 RA) in people living with HIV and T2D is sparse. Only one prospective (24 weeks) observational study reported the experience with the SGLT2i canagliflozin in 8 HIV-infected diabetic subjects (11), and only three case reports have so far described the use of GLP-1 RA (12–14). Then, our case reporting on a lady with T2D and ART-treated HIV in whom the treatment with dulaglutide ensured clinically significant improvement of glycemic control, treatment adherence with no hypoglycaemia adds up to the existing literature to support the feasibility of using GLP-1 RAs in these subjects. Of interest, our case also had high cardiovascular (CV) risk as indicated by multidistrict atherosclerotic disease (carotid, renal and cerebral arteries), a condition where the use of a GLP1-RA with established CV benefit is highly recommended (9, 10). This is even more critical on the light that ASCVD remains a main cause of morbidity and mortality among PLWH (2) to the point that the American Heart Association has recently recommended considering HIV as a major CV risk factor (15). The reasons for an elevated risk of ASCVD among people living with HIV remains relatively poorly understood, but they are likely to be multifactorial (16). In the absence of randomized clinical trials or hypothesis-testing mechanistic studies, it is only possible to speculate that GLP-1 RAs can contribute to reduce the increased CV risk in subjects with HIV *via* “glycemic” and “extra- glycemic” actions including a beneficial effect on blood pressure, lipid profile, body fat, insulin resistance, and inflammation among the many ones (17).

On the other hand, to the best of our knowledge, cardiovascular outcome trials (CVOTs) investigating the safety and efficacy of GLP-1RAs did not specifically report HIV infection as an inclusion or exclusion criterion and no analyses or sub-analyses on this specific subgroup of patients have been reported out of these CVOTs (18–25). The management of T2D in people with HIV is still a matter of discussion (8) and subjects with HIV pose a special challenge because of possible drug interactions. Integrase strand inhibitors can increase the area under the curve of metformin. Though this may not be clinically significant (26) it may still require some caution (27). Of interest, a recent study showed that HIV-1 replication is suppressed by metformin in both primary human CD4 T cells and humanized mice (28), which may open new opportunities in the future. Some protease inhibitors are CYP2C9 inducers and can decrease sulfonylurea levels (29, 30). Conversely, pioglitazone has been suggested to be the drug of choice in HIV-1-infected lipoatrophic adult individuals, although more data are needed on the overall safety of this compound (31). Moreover, when used along with CYP2C8 inhibitors (many protease inhibitors) its circulating levels may increase (32). Concern regarding gliptin use in HIV-infected individuals has been raised, as these agents have molecular targets on immune cells, although a small study showed no changes in CD4 or HIV RNA among treated HIV-infected subjects on sitagliptin (33). Of note, saxagliptin has been showed to interact with CYP3A4/5 inhibitors such as atazanavir, indinavir, ritonavir, and saquinavir (30, 32). No interactions between ART and SGLT-2 inhibitors are expected; however, UDP-glucuronosyltransferase enzyme inducers (e.g., ritonavir) may decrease the exposure to canagliflozin (32). To the best of our knowledge, no interactions between ART and GLP-1 RAs have been reported. In a patient like the one described here, the use of a GLP1-RA appears to be rational enough. SGLT2 inhibitors could have been a choice in our patient as well. However, the presence of a mycotic infection at hospital admission suggested a safer use of GLP-1 RA in this subject and dulaglutide proved to have a positive impact on the achievement and maintenance of glycemic control. Moreover, its weekly administration along with no occurrence of hypoglycemic events may have accounted for a good treatment adherence in a person who had in the past much trouble in managing a more complex insulin treatment. Also, the use of dulaglutide had no negative effects on liver or renal function. On possible matter of concern could have been the possible effect of delayed gastric emptying of GLP-1 agonists and the possible impact on orally administered oral agents. However, the gastric effect of long acting GLP-1 RAs like dulaglutide tends to vanish with time because of a tachyphylaxis. Moreover, dulaglutide has been shown not to alter bioavailability of many commonly used oral agents (34). As a possible downside, a 3 kg body weight reduction was recorded. Although this may be looked at with some concern, the reduction in body weight obtained with GLP-1 RAs, more evident within the first month of therapy, tends to plateau from the first to the 5th year of therapy (23) so that no further loss is expected with the prosecution of the therapy. Rather, this weight loss may have a favorable impact of ectopic fat and, in particular, on liver fat that tends to accumulate in response to antiretroviral therapy (35). Our subjects also had a renal artery stenosis and mild hypertension. With respect to this too, a GLP1-RA can be considered an interesting therapeutic option due to the known effect of these medications on blood pressure and because of pre-clinical data suggesting they could induce a vasodilation of the renal arteries (36).

In conclusion, our experience, although limited to a single case report, suggests that GLP-1 RA therapy may be a valid therapeutic opportunity in people living with HIV. Our observation calls for proper clinical studies exploring the potential use of GLP-1 RA in patients with special forms of diabetes or diabetes associated with special clinical condition such as HIV and its anti-viral treatment. To this extent, two randomized clinical trials are currently investigating the potential of GLP-1 RAs in these individuals. The first study will assess the efficacy of semaglutide as an adjunct to diet and exercise in achieving greater weight loss as compared to diet and exercise alone in HIV-1 infected patients ≥ 18 years with BMI ≥ 30 kg/m2 or BMI ≥ 27 kg/m2 and hypertension, dyslipidemia or type 2 diabetes (ClinicalTrials.gov Identifier: NCT04174755). The second trial will evaluate the efficacy of semaglutide vs placebo in treating lipohypertrophy among non-diabetic people living with HIV (ClinicalTrials.gov Identifier: NCT04019197).

## Data Availability Statement

The raw data supporting the conclusions of this article will be made available by the authors, without undue reservation.

## Ethics Statement

Ethical review and approval were not required for a case report study in accordance with the local legislation and institutional requirements. The patient provided the written informed consent to partecipate in this study.

## Author Contributions

AD conceived the case report description and wrote the first draft. AD and MA were involved in the clinical management of the patient. AD, GD, and RM made a critical literature review. SDP made a critical interpretation of the intellectual content of the article. AD is the guarantor of this work and, as such, had full access to all the data in the study and takes responsibility for the integrity of the data and the accuracy of the data analysis. All authors contributed to the article and approved the submitted version.

## Conflict of Interest

The authors declare that the research was conducted in the absence of any commercial or financial relationships that could be construed as a potential conflict of interest.

## Publisher’s Note

All claims expressed in this article are solely those of the authors and do not necessarily represent those of their affiliated organizations, or those of the publisher, the editors and the reviewers. Any product that may be evaluated in this article, or claim that may be made by its manufacturer, is not guaranteed or endorsed by the publisher.
